# Complete Genome Assemblies of Three Highly Prevalent, Toxigenic Clostridioides difficile Strains Causing Health Care-Associated Infections in Australia

**DOI:** 10.1128/MRA.00599-21

**Published:** 2021-08-05

**Authors:** Keeley O’Grady, Thomas V. Riley, Daniel R. Knight

**Affiliations:** a Centre for Biosecurity and One Health, Harry Butler Institute, Murdoch University, Murdoch, Western Australia, Australia; b School of Biomedical Sciences, The University of Western Australia, Queen Elizabeth II Medical Centre, Nedlands, Western Australia, Australia; c School of Medical and Health Sciences, Edith Cowan University, Joondalup, Western Australia, Australia; d Department of Microbiology, PathWest Laboratory Medicine, Queen Elizabeth II Medical Centre, Nedlands, Western Australia, Australia; University of Maryland School of Medicine

## Abstract

Clostridioides difficile infection (CDI) is the leading cause of life-threatening health care-related gastrointestinal illness worldwide. Phylogenetically appropriate closed reference genomes are essential for studies of C. difficile transmission and evolution. Here, we provide high-quality complete hybrid genome assemblies for the three most prevalent C. difficile strains causing CDI in Australia.

## ANNOUNCEMENT

Clostridioides difficile causes life-threatening diarrhea and health care-related gastrointestinal infections globally (1). Core genome single nucleotide polymorphism (cgSNP) analysis is highly discriminatory for bacterial transmission and outbreak detection studies and the gold standard for reconstructing large phylogenies of closely related microbes (2). A critical step in cgSNP analysis involves mapping raw sequence data to a closely related reference genome, allowing for variant sites to be identified, filtered, and compared between strains (3). Using phylogenetically related “closed” reference genomes provides optimal mapping and variant calling. Australia has a diverse C. difficile population distinct from that of the rest of the world (1, 4), yet there are no phylogenetically appropriate reference genomes. Here, combining short- and long-read sequence technologies, we provide high-quality complete genome sequences for three of the most prevalent C. difficile strains causing C. difficile infection (CDI) in Australia, PCR ribotype 014 (RT014) (29.5% prevalence), RT002 (11.8%), and RT056 (5.4%) (5, 6). Representative C. difficile strains of each ribotype (S-0352, S-0253, and S-0942, respectively) were selected from >1,500 isolates recovered from patients with symptomatic CDI, part of the ongoing nationwide longitudinal surveillance of CDI in Australia, the C. difficile Antimicrobial Resistance Surveillance (CDARS) study (5, 6).

C. difficile strains from CDARS were cultured on blood agar in an anaerobic chamber (80% N_2_, 10% CO_2_, 10% H_2_) for 48 h (5). Total genomic DNA was extracted using a QuickGene DNA tissue kit (Kurabo Industries, Osaka, Japan) and used as input for both short-read (Illumina) and long-read (Oxford Nanopore Technologies [ONT]) sequencing. Illumina whole-genome sequencing (WGS) was performed using standard Nextera Flex paired-end read (2 × 150-bp) libraries on an Illumina NovaSeq 6000 instrument (Illumina, San Diego, CA, USA) to an average read depth of 130×. Default parameters were used for all software unless specified. The raw reads were filtered for quality (Q30+) and adaptor sequences using Trim Galore v0.6.5 (https://github.com/FelixKrueger/TrimGalore). ONT sequencing was performed on a MinION Mk1C device (ONT, Oxford, UK) using an R9 generation flow cell following a DNA by ligation protocol (SQK-LSK109). Filtlong v0.2.0 (https://github.com/rrwick/Filtlong) was used to filter the low-quality reads (keeping the top 90% of reads and removing reads of <1,000 bp), resulting in 2.28 (S-0352), 2.59 (S-0253), and 6.66 (S-0942) Gb of sequence data, respectively. The hybrid assembly of ONT and Illumina reads was performed using Unicycler v0.4.8 (7) with multiple rounds of polishing (Pilon v1.2.4, Racon v1.4.3) to improve the contiguity. Complete circular genomes were confirmed using Bandage v0.8.1 (8) and rotated to *dnaA* using Unicycler. The genomes were evaluated using QUAST v2.344 (http://quast.sourceforge.net/quast) and annotated using the NCBI Prokaryotic Genome Annotation Pipeline v5.2 (9). The multilocus sequence type (ST) was determined using PubMLST (10).

The summary genome features and metrics are shown in Table 1. A single 6,760-bp plasmid was identified in S-0253 (RT002). This data set increases the diversity of complete reference genomes available to the C. difficile research community, aiding future studies of C. difficile transmission and evolution.

**TABLE 1:** Key features of C. difficile genomes

Feature	Data for strain:		
	S-0352	S-0253	S-0942
Strain epidemiology^*a*^	RT014, ST2, clade 1	RT002, ST8, clade 1	RT056, ST34, clade 1
Toxin profile^*b*^	A^+^B^+^CDT^−^	A^+^B^+^CDT^−^	A^+^B^+^CDT^−^
Origin^*c*^	Human, CDI, VIC 2014	Human, CDI, SA 2014	Human, CDI, SA 2016
GenBank accession no.	CP076377	CP076401, CP076402	CP076376
ENA accession no.	ERS5447138	ERS5447236	ERS5447376
Genome size (bp)	4,251,987	4,089,134 (4,095,894^*d*^)	4,129,159
%GC	28.96	28.52	28.71
No. of CDS^*e*^	3,790	3,591	3,648
No. of contigs	1	2	1
No. of tRNAs	90	90	90
No. of rRNAs	35	35	35
No. of CRISPRs^*f*^	10	4	9
Read metrics			
Total no. of ONT reads	545,760	760,250	1,400,000
Average ONT read length (bp, filtered)	5,518	4,663	9,038
Total no. of Illumina reads (trimmed)	2,015,674	1,930,928	1,868,362

a RT, PCR ribotype; ST, multilocus sequence type.

b Presence/absence of full-length *tcdA*, *tcdB* (pathogenicity locus, PaLoc), and binary toxin *cdtA/B* (binary toxin locus, CdtLoc). CDT, C. difficile binary toxin.

c VIC, Victoria; SA, South Australia,

d Combined chromosome and plasmid length.

e CDS, coding sequences.

f CRISPRs, clustered regularly interspaced short palindromic repeats.

### Data availability.

The genome data are available at GenBank under BioProject accession number PRJNA734443 (complete genome assemblies) and at the ENA under BioProject accession number PRJEB41588 (Illumina sequence data); see Table 1 for details.
